# Cardiomyocyte Circadian Oscillations Are Cell-Autonomous, Amplified by β-Adrenergic Signaling, and Synchronized in Cardiac Ventricle Tissue

**DOI:** 10.1371/journal.pone.0159618

**Published:** 2016-07-26

**Authors:** Stephen Beesley, Takako Noguchi, David K. Welsh

**Affiliations:** 1 Center for Circadian Biology, University of California San Diego, La Jolla, California, United States of America; 2 Department of Psychiatry, University of California San Diego, La Jolla, California, United States of America; 3 Veterans Affairs San Diego Healthcare System, San Diego, California, United States of America; Kent State University, UNITED STATES

## Abstract

Circadian clocks impact vital cardiac parameters such as blood pressure and heart rate, and adverse cardiac events such as myocardial infarction and sudden cardiac death. In mammals, the central circadian pacemaker, located in the suprachiasmatic nucleus of the hypothalamus, synchronizes cellular circadian clocks in the heart and many other tissues throughout the body. Cardiac ventricle explants maintain autonomous contractions and robust circadian oscillations of clock gene expression in culture. In the present study, we examined the relationship between intrinsic myocardial function and circadian rhythms in cultures from mouse heart. We cultured ventricular explants or dispersed cardiomyocytes from neonatal mice expressing a PER2::LUC bioluminescent reporter of circadian clock gene expression. We found that isoproterenol, a β-adrenoceptor agonist known to increase heart rate and contractility, also amplifies PER2 circadian rhythms in ventricular explants. We found robust, cell-autonomous PER2 circadian rhythms in dispersed cardiomyocytes. Single-cell rhythms were initially synchronized in ventricular explants but desynchronized in dispersed cells. In addition, we developed a method for long-term, simultaneous monitoring of clock gene expression, contraction rate, and basal intracellular Ca^2+^ level in cardiomyocytes using PER2::LUC in combination with GCaMP3, a genetically encoded fluorescent Ca^2+^ reporter. In contrast to robust PER2 circadian rhythms in cardiomyocytes, we detected no rhythms in contraction rate and only weak rhythms in basal Ca^2+^ level. In summary, we found that PER2 circadian rhythms of cardiomyocytes are cell-autonomous, amplified by adrenergic signaling, and synchronized by intercellular communication in ventricle explants, but we detected no robust circadian rhythms in contraction rate or basal Ca^2+^.

## Introduction

Endogenous daily oscillators with periods near 24 hrs, known as circadian clocks, have evolved in living organisms to anticipate environmental day/night cycles. These daily oscillators regulate the timing of many physiological processes, such as sleep/wake cycles, metabolism, hormonal activity, and heart rate [1, 2]. Circadian clocks exist throughout phylogeny, from prokaryotic cyanobacteria to mammals. This evolutionary conservation suggests that circadian clocks are of critical importance for survival. In the mammalian brain, the suprachiasmatic nucleus (SCN) is the central oscillator, and is synchronized to the external environment by light, via the retina. Peripheral cells, too, contain circadian oscillators: tissues such as heart, lung, liver, and kidney, as well as primary fibroblasts and various immortalized cell lines, remain rhythmic in culture for many days [2–4].

Within single cells, the circadian oscillator consists of a molecular feedback loop. BMAL1 and CLOCK (or NPAS2) form complexes that bind to DNA at E-box motifs and initiate transcription of *Period (Per)* and *Cryptochrome (Cry)* genes [5]. PER1/2 and CRY1/2 are transcriptional repressors, which subject to delays at each transcription and translation step, form complexes in the cytoplasm, translocate to the nucleus, and repress BMAL1/CLOCK activity [6, 7]. Importantly, rhythmic PER2 is essential for circadian oscillations [8], acting as the bridge between CRY1 and the BMAL1/CLOCK [9]. This inhibitory complex is eventually degraded via the proteasome, through casein kinase 1δ/ε (CK 1δ/ε) phosphorylation [10] and F-box protein ubiquitination, resulting in initiation of the next circadian cycle [11].

Approximately 10% of the heart transcriptome is under circadian control [12, 13], and there is a dramatic time of day bias for pathological events such as myocardial infarction, stroke, and sudden cardiac death [14–16]. Although this important circadian modulation is synchronized by the central SCN circadian pacemaker via sympathoadrenal activity, it is probably driven at least in part by circadian clocks intrinsic to the heart itself. The importance of local circadian clocks has been elegantly demonstrated for rhythmic gene transcription in the liver [17] and electrophysiology in the retina [18]. Heart rate and blood pressure are under circadian control even independent of locomotor activity [19], suggesting the possibility of control by circadian clocks in the heart. Pioneering studies have demonstrated circadian clocks intrinsic to adult rat heart tissue and dispersed cardiomyocytes using RT-PCR [20] and in cultured arteries, veins, and ventricle explants from adult mice bearing a *Per1* promoter-driven luciferase reporter [21]. However, no prior studies have addressed circadian rhythms in neonatal heart or how they are modulated by tissue organization or cell signaling.

The importance of a functional molecular clock within the heart has been demonstrated using various clock gene mutant mice. The core circadian clock protein CLOCK is enriched in the heart compared to other peripheral tissues [22], and cardiomyocyte-specific *Clock-*mutant mice lack diurnal oscillations in expression of ion channel subunit proteins (e.g. the voltage-gated calcium channel VGCC1α1D) and in phosphorylation of several kinases (e.g. extracellular-signal-regulated kinases, ERK) [23]. In the *Bmal1* whole body knock-out mouse, the heart is severely affected, with smaller cardiomyocytes, thinning of the myocardial wall, and disruption of sarcomeres [24]. Further studies using an inducible cardiomyocyte-specific *Bmal1* knock-out mouse have revealed that circadian expression of several cardiac ion channels (Kcnh2 and NAv1.5) requires *Bmal1* expression intrinsic to cardiomyocytes [25, 26]. Highly regulated control of Ca^2+^ underlies many essential cellular processes. Particularly in the heart, where a highly coordinated, rhythmic release and reuptake of Ca^2+^ from sarcoplasmic reticulum across millions of cells underlies each heartbeat [27]. Interestingly, a previous study reported that intracellular Ca^2+^ concentration is higher in rat cardiomyocytes isolated during the day vs. night [28], suggesting the existence of circadian Ca^2+^ regulation *in vivo* that persists even in freshly isolated cardiomyocytes.

Clear circadian oscillation of Ca^2+^ has been demonstrated in the SCN [29, 30], and we hypothesized that Ca^2+^ is also under circadian control in the heart, and detectable with the genetically encoded fluorescent Ca^2+^ reporter GCaMP3. To study the relationship between cardiac function and circadian rhythms, we tested the effect of isoproterenol on clock gene rhythms in heart tissue from PER2::LUC reporter mice. To determine if interactions among cardiomyocytes within the highly organized tissue structure of ventricle explants play a role in robust circadian rhythm generation, we directly compared circadian rhythms in dispersed cardiomyocytes and ventricular explants from neonatal mice by single-cell PER2::LUC imaging. Furthermore, we report the development of an imaging method for simultaneous, long-term recording of PER2::LUC bioluminescence and GCaMP3 fluorescence in single cardiomyocytes, permitting direct comparison of PER2 and Ca^2+^ data.

## Methods

### Animals

Generation of mPer2^Luciferase^ (PER2::LUC) knockin mice was described previously [31]. For this study, we used an alternative PER2::LUC mouse line incorporating an SV40 polyadenylation site to enhance expression levels [32]. The mice were developed at Northwestern University using the same methodology as the original strain of knockin mice [33]. A mouse line expressing both GCaMP3 and PER2::LUC (GCaMP3-PER2::LUC) was generated by crossing a mouse expressing GCaMP3 in a Cre-dependent manner (Jackson Laboratory, Stock #014538, Sacramento, CA) with a ZP3-cre mouse (Jackson Laboratory Stock #003651) in which Cre is expressed in 100% of oocytes, and then crossing to the PER2::LUC mouse. PER2::LUC mice were bred as homozygotes and maintained in LD 12:12 light cycles (12 hr light, 12 hr dark) throughout gestation and from birth until used for experiments. GCaMP3 mice were bred as heterozygotes. This study was carried out in strict accordance with the recommendations in the Guide for the Care and Use of Laboratory Animals of the National Institutes of Health. The protocol was approved by the UCSD Institutional Animal Care and Use Committee at University of California, San Diego (Animal Use Protocol S07365). Neonatal mice were anesthetized by intraperitoneal injection of ketamine (Fort Dodge Animal Health, Fort Dodge, IA) before decapitation. All efforts were made to minimize animal suffering.

### Tissue explants

**Fig 1:**
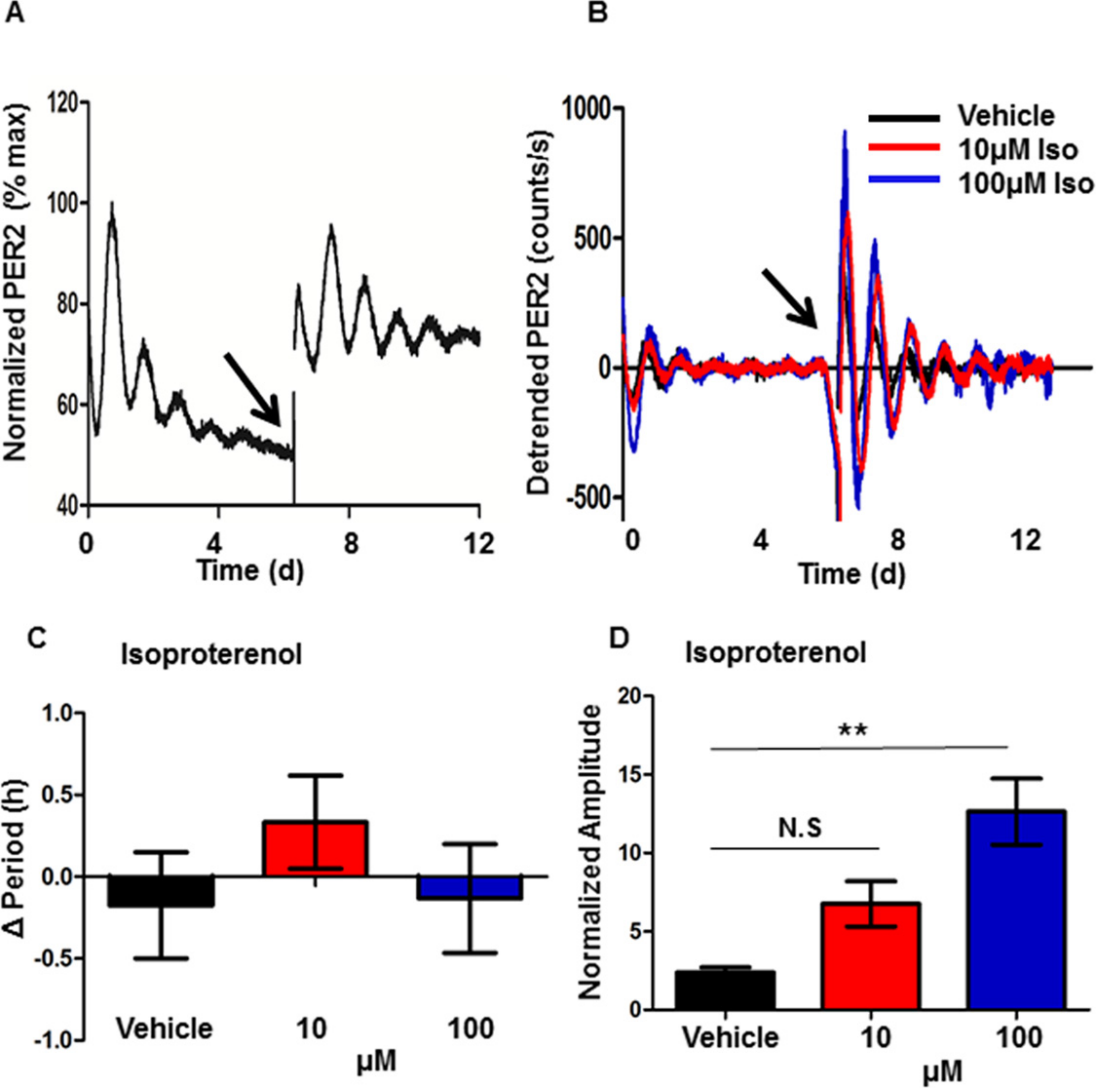
β-adrenergic receptor activation increases PER2::LUC rhythm amplitude in ventricle explants. (A-B) Neonatal ventricle slices were synchronized with serum, and PER2::LUC rhythms were recorded for 2 weeks. At 7 days, medium was exchanged for medium containing either drug or vehicle control (arrow). (A) Representative PER2::LUC oscillations are shown for a control slice, expressed as a percentage of maximum bioluminescence intensity. (B) Representative detrended PER2::LUC oscillations are shown for slices treated with either vehicle (black trace), 10 μM isoproterenol (Iso) (red trace), or 100 μM Iso (blue trace), expressed as bioluminescence intensity in counts/sec, with linear trend subtracted. The arrow shows the time of drug or vehicle treatment. Change in circadian rhythm period (C) and normalized amplitude (second peak, relative to initial serum treatment) (D) are shown for each drug treatment condition. Statistical analysis was by one-way ANOVA with a Bonferroni post hoc correction, ** *p* < 0.01.

**Fig 2:**
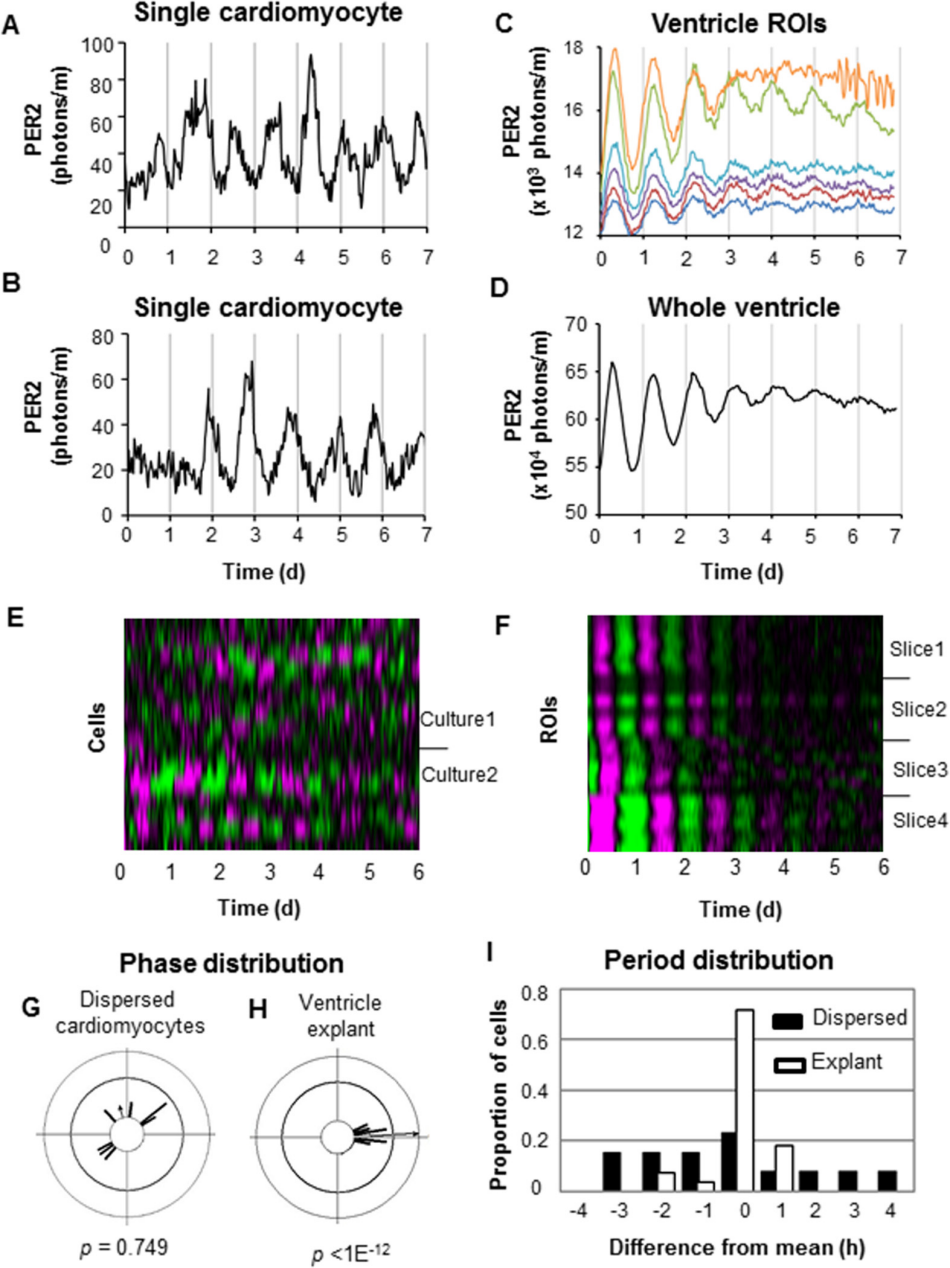
Imaging of PER2::LUC rhythms in dispersed single cardiomyocytes and cell-sized regions of ventricular tissue. Dispersed PER2::LUC cardiomyocytes or 300 μm ventricular explant cultures were placed under a CCD camera and imaged for 6–7 days. (A- B) PER2::LUC oscillations of two single cardiomyocytes in the same dispersed culture. (C-D) PER2::LUC oscillations of randomly placed cell-sized regions of interest (ROIs) in a ventricle explant (C) and of a whole ventricle (D). (E-F) Raster plots depicting PER2::LUC oscillations of 16 single cardiomyocytes in 2 dispersed cultures (9 in culture 1, 7 in culture 2) (E) and ROIs from 4 ventricular slices (n = 7 ROIs per slice) (F), with higher than average bioluminescence intensity coded by magenta and lower than average intensity by green. Each row is a single cell (E) or ROI (F). In E, cells were ordered by the time of peak in the interval 0.6–1.6 d. In F, ROI’s were ordered randomly. Time is relative to start of recording. (G-H) Phase distributions of dispersed cardiomyocytes (G) and regions in a representative explant (H) are shown as Rayleigh plots. *p*-values are from Rayleigh tests for synchrony. (I) Distribution of periods of dispersed cardiomyocytes and explant culture ROIs. Proportions of all 13 rhythmic single cardiomyocytes or of all 28 cell-sized ROIs are shown on the y-axis. Differences from the mean period within a culture are shown on the x-axis. Filled black bars represent dispersed cardiomyocytes. Open white bars represent ROIs in ventricle explants.

Hearts were removed from 2 day old PER2::LUC mice (for dispersed cardiomyocyte cultures) or GCaMP3-PER2::LUC mice (for dispersed cardiomyocyte culture and ventricular explants) and placed directly into ice-cold Ca^2+^- and Mg^2+^-free Hank’s Balanced Salt Solution (HBSS) (Thermo Fisher Scientific 14175–095, Waltham, MA) for 10 minutes, and squeezed gently to remove blood. Hearts were transferred to fresh ice-cold HBSS, and the atria were removed. Each ventricle was then cut into small sections and placed directly onto a membrane insert (organotypic inserts PICM0RG50, Millipore, Billerica, MA) in a 35 mm dish containing luminescence recording medium, as described previously [34]. For imaging, ventricles were embedded in 1% agarose, 300 μm slices were cut using a cooled vibratome (VT1200S, Leica, Nussloch, Germany), and the resulting slices were placed on a membrane in explant medium: Dulbecco’s Modified Eagle’s Medium (DMEM) (Mediatech 90-013-PC, Manassas, VA), serum-free, 0.35 g/L sodium bicarbonate, without phenol red, pH 7.4, supplemented with 10 mM HEPES, 25 U/ml penicillin, 25 μg/ml streptomycin, and 2% B-27 (Thermo Fisher Scientific 17504–044). SCN slices were obtained from 4–6 day-old PER2::LUC mice as described previously [35] and were transduced with an AAV2/1-hsynapsin-GCaMP3 viral vector (Penn Vector Core, University of Pennsylvania). The dish was sealed with a 40 mm circular coverslip (40CIR1, Erie Scientific, Portsmouth, NH) and kept at 37°C without CO_2_ until transfer to the luminometer (LumiCycle, Actimetrics, Wilmette, IL). Prior to recording ventricle explants, circadian rhythms were synchronized with 50% horse serum for 1 hour, then washed in HBSS and returned to fresh recording medium. Seven days after synchronization, to increase heart rate, some slices were treated with isoproterenol (Sigma-Aldrich, St. Louis, MO) dissolved in ethanol (Figs 1 and 2, slices 1–2). For drug experiments, existing medium was removed and fresh medium with vehicle or drug was added. Synchronization with 50% horse serum was not performed for non-beating ventricle explants (Figs 2, slices 3–4, and 3F–3G).

### Primary cardiomyocyte isolation

Cardiomyocytes were isolated as in Sreejit et al. (2008) [36], with some modifications. Ventricles from 2 day old PER2::LUC mice or GCaMP3-PER2::LUC mice were dissected as described above, pooled, and lightly minced. Tissue was pre-digested in 0.5% trypsin (Thermo Fisher Scientific 15090–046) in Ca^2+^- and Mg^2+^-free HBSS, overnight at 4°C. The following day, 50 mg/ml trypsin inhibitor (Sigma-Aldrich) was added and the tissue warmed to 37°C. Collagenase Type 1 (Worthington, Lakewood, NJ) at 1400 Units/ml, dissolved in Liebovitz medium (Thermo Fisher Scientific, no antibiotics or serum), was added, and the tissue was incubated at 37°C for 40 minutes. Dissociation was completed by repeated pipetting and passage through a 100 μm mesh (Cell Strainers, Thermo Fisher Scientific). The solution was centrifuged at 500 x g for 5 minutes and re-suspended in DMEM supplemented with 10% fetal bovine serum (FBS) (Thermo Fisher Scientific, 26140–079), 25 U/ml penicillin, and 25 μg/ml streptomycin. To remove fibroblasts, cells were pre-plated twice for 1 hr on plates coated with 1% collagen (Thermo Fisher Scientific, A10483-1). Cells were then centrifuged and re-suspended in DMEM, supplemented as previously, and plated onto 35 mm glass bottom dishes (MatTek, Ashland, MA) coated with 1% collagen. After incubation at 37°C, 5% CO_2_, for 2 days, fresh DMEM was added with 1 μM arabinofuranosyl cytidine (AraC, Sigma-Aldrich, C6645), to inhibit DNA synthesis and proliferation of contaminating fibroblasts. At 48 hrs before luminometry or imaging, the cells prepared from PER2::LUC mice were transduced with an AAV2-*pCMV*-*GCaMP3* Ca^2+^ reporter, using approximately 2 x 10^7^ infection units. (The plasmid was a gift of Dr. Loren Looger at Janelia Farm [37]. AAV vector was produced by University of California, San Diego core virus facility). For luminometry or imaging, cells were washed in HBSS and then placed in luminescence recording medium: DMEM (Mediatech), serum-free, 0.35 g/L sodium bicarbonate, without phenol red, pH 7.4, supplemented with 10 mM HEPES, 25 U/ml penicillin, 25 μg/ml streptomycin, and 2% B-27 (Life Technologies), 0.2 mM luciferin potassium salt (BioSynth L-8220, Staad, Switzerland), and the dish was sealed.

### Immunohistochemistry

For immunofluorescence experiments, dissociated cardiomyocytes were seeded at 2.6 x 10^5^ cells/cm^2^ onto a glass coverslip, and left to adhere overnight. The cells were then washed in phosphate buffered saline (PBS) and fixed with 3% paraformaldehyde. Cells were again washed in PBS, permeabilized for 4 minutes with 0.1% Triton X-100 in PBS, and washed again. The primary antibody, mouse anti-alpha actinin (Sigma-Aldrich), was diluted 1:500 in PBS with 0.5 mg/ml bovine serum albumin (BSA) and added to the cells for 20 minutes. After washing, the secondary antibody, Texas red labelled goat anti-mouse IgG (Vector labs) diluted 1:200 in PBS with 0.5 mg/ml BSA was applied for a further 20 minutes at room temperature. The cells were again washed in PBS, and the cover slips were mounted on glass slides and fixed with VectaShield mounting medium (Vector Labs) containing 4',6-diamidino-2-phenylindole (DAPI) for nuclear staining. Cells were imaged and analyzed using ImageJ (NIH).

### Short-term video recording

To identify cardiomyocytes, beating was observed in bright field (BF) or GCaMP3 fluorescence (FL). The culture was placed on the stage of an inverted microscope (Olympus IX-71, Tokyo, Japan). Light source was TH4-100 (Olympus) for BF, and X-Cite series 120 (EXFO, Quebec, Canada) for FL. Light from the sample was collected by a 40x objective lens (LUCPlanFLN 40x; Olympus) and transmitted to a CCD (charge-coupled device) camera (DP72, Olympus) mounted on the side port of the microscope. The movies were produced using BF imaging (S1 Movie), and a GFP filter cube (GFP-3035B-OMF-ZERO; Semrock, New York, NY) with 10x magnification (CPlanFLN 10x, Olympus) for the Ca^2+^ flashes (S2 Movie).

### Simultaneous clock gene, basal Ca^2+^ level, and contraction rate imaging

**Fig 3:**
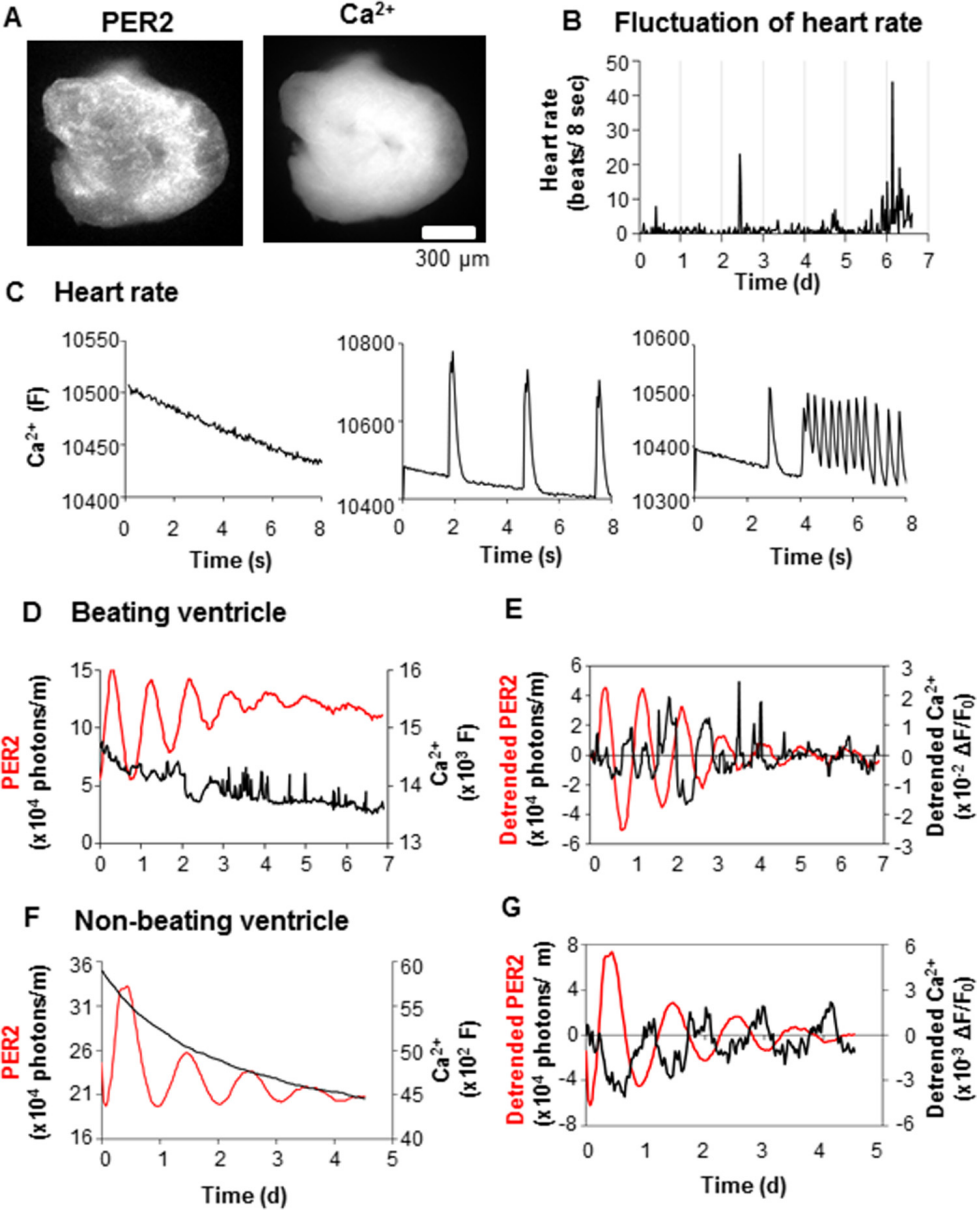
Simultaneous monitoring of PER2 level, Ca^2+^ level, and spontaneous heartbeat rate in cultured ventricle explants. (A) Representative PER2::LUC and Ca^2+^ images of ventricle explants. (B) Representative fluctuation of the myocardial contraction rate of a ventricle explant. The number of beats was determined every 30 min for >6 days by Ca^2+^ imaging at 24 fps for an 8 s video stream at each time point. (C) Representative traces of Ca^2+^ surge activity recorded by stream video of beating ventricle explants. Left, middle, and right examples are 0, 3, and 13 beats per 8 s, respectively. Y-axis is raw fluorescence intensity, x-axis is time after start of the stream video. (D-G) Representative traces of PER2::LUC and basal Ca^2+^ (average Ca^2+^ level for 1 s) from beating (D-E) and (F-G) non-beating ventricle explants. Traces shown in D and F are raw data, and E and G are detrended by subtracting a 24 h running average. PER2::LUC and Ca^2+^ traces are shown in red and black lines, respectively. Times in B and D-G are after start of recording.

Clock gene expression was monitored by PER2::LUC bioluminescence (BL). Basal Ca^2+^ level and contraction rate were monitored by GCaMP3 FL. The system was developed by adding FL imaging to previously described methods for BL imaging [38]. In a dark room, stray light was eliminated by covering the dish with a small black lucite box and draping the microscope with black plastic sheeting (Thorlabs BK5, Newton, NJ). The culture was placed on the stage of an inverted microscope (Olympus IX-71). Light from the sample was collected by either a 10x or 4x objective lens (10x UPlanSApo, 4x XLFLUOR; Olympus) and transmitted to a cooled CCD camera (iKon-M; Andor, Belfast, UK) mounted on the bottom port of the microscope. The image was focused using GCaMP3 FL. GCaMP3 was excited by a 500 nm LED (light-emitting diode) (pE-2; coolLED, Andover, UK) and emitted light was filtered by a yellow fluorescent protein (YFP) filter cube (YFP-2427A; Semrock, Rochester, NY). To avoid autofluorescence of luciferin, excitation and filter settings were optimized for yellow fluorescent protein (YFP) rather than for green fluorescent protein (GFP), which has absorbance and emission spectra similar to GCaMP3 [23]. LED light intensity was reduced to either 10% or 50% of maximal level, depending on GCaMP3 expression levels. To assess basal Ca^2+^ level, FL images were collected at intervals of 30 min with 1 s exposure duration, no binning, pre-amplifier gain x 4, and readout speed 5 MHz (Fig 3D–3G). FL imaging was limited to 1 s every 30 min because it could only be performed between successive BL images, each requiring 29 min exposure time. To monitor contraction rate, a video stream was taken every 30 min, for 8 s, with 20 ms exposure time per frame x 200 frames, with LED intensity 5% of maximal level. Duration of the stream video was limited to 8 s because a declining baseline FL was observed due to photobleaching of GCaMP3 (Fig 3C, 3D and 3F). Sizes of stream images were reduced by restricting acquisition to the central 64 x 64 pixels (center quad function), and then binning pixels 8 x 8. During video streaming, the camera shutter was always open (Fig 3C). For BL image collection, the LED light was turned off, and the filter cube turret was moved to an open (no filter cube) position. BL images were collected at intervals of 30 min, with 29 min exposure duration, binning 1 x 1 or 4 x 4, pre-amplifier gain x 4, and readout speed 50 kHz, alternating with FL images and streaming. To align with FL images, BL images were shifted 1 pixel in the *x* direction and -9 pixels in the *y* direction. MetaMorph (Molecular Devices, Sunnyvale, CA) was used for control of camera and LED shutter, and for image analysis. Time series images were typically collected for 5–7 days.

### Image processing

**Fig 4:**
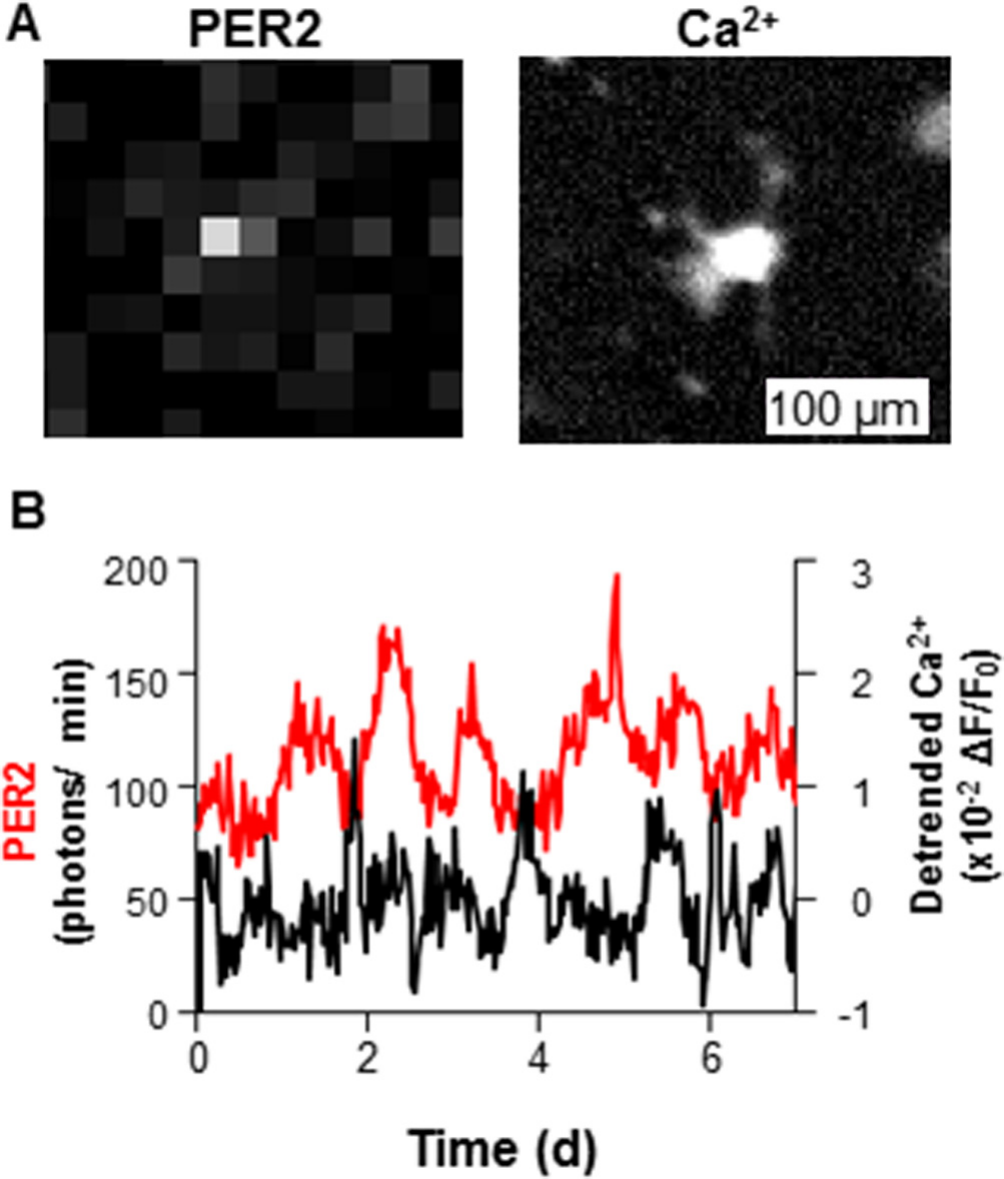
Simultaneous monitoring of basal Ca^2+^ and PER2 levels in dispersed cardiomyocytes. (A) Representative PER2::LUC (left) and GCaMP3 (right) images of a single cardiomyocyte in dispersed culture. (B) Patterns of Ca^2+^ (GCaMP3, black) and PER2 (PER2::LUC, red) of the cardiomyocyte shown in a recorded over 7d.

In MetaMorph, cosmic ray artifacts were removed from BL images by using the minimum value for each pixel in a pixel-wise comparison of two consecutive images. Thus, the data were effectively smoothed by a running minimum algorithm, using a 1 hr temporal window. The resulting images were stacked to give a series of consecutive images. Cosmic ray removal was not necessary for GCaMP3 FL images. To determine which cells were cardiomyocytes or contaminating fibroblasts in dispersed cell culture, we used the GCaMP3 FL images (Figs 2 and 4). Cells that were seen to be ‘flashing’ by direct eye observation of GCaMP3 FL images were identified as cardiomyocytes and marked with a region of interest (ROI). The ROI was then transferred to the same location on the BL image stack. The BL and FL intensities were measured within the ROI for each identified cell and each time point. The position of the ROI was adjusted if necessary to accommodate movements of cells. The identification of single cells was difficult in images of ventricle explants, so we used single-cell sized ROIs (Figs 2 and 3). ROIs of sizes similar to those used for single cells in dispersed cell cultures were randomly placed across ventricle explants. Data were logged to Microsoft Excel (Microsoft, Redmond, WA) for further processing and plotting. BL intensity values were converted to photons/min based on the rated quantum efficiency and gain of the camera. Raw FL intensity (F) is shown in Fig 3C, 3D and 3F. To remove long-term baseline fluctuations, data were detrended by subtracting a 24 h running average from either BL intensity or FL change relative to initial FL intensity (F_0_) (ΔF/F_0_) (Figs 3E, 3G, and 4B). The magnitude of fluctuation of basal Ca^2+^ level relative to background FL level was defined as the difference between the maximum and minimum values of detrended data in the range from 0.5 d after start of recording to the end of recording. Rhythm parameter analysis was performed using LumiCycle Analysis software (Actimetrics, Wilmette, IL). The best fit sine curve was obtained using LM Fit (Damped sin) to find the set of parameters that gives best least-squares fit of data to the equation: Y(t) = A*sin(2πft+φ)*e^-t/τ^+C, where t is time, A is amplitude, f is frequency, φ is phase, τ is damping tau, and C is offset. Circular phase graphs generated by Oriana (Kovach Computing Services, Anglesey, UK) (Fig 2G and 2H) were used to show the phase of the peak occurring 0.6–1.6 d after start of recording. Coherence of phases was tested by Rayleigh test using Oriana.

### Analysis of luminometer data

Circadian oscillations of ventricle explants were normalized to the vehicle control, for which the first PER2::LUC luminescence peak after synchronization was assigned a value of 100 (Fig 1A). For baseline-subtracted traces (Fig 1B), long-term trends were removed by subtracting a 24 hr moving average. For drug treatment experiments (Fig 1D), amplitude was defined as the peak intensity of the second oscillation after drug application, relative to the second peak after synchronization with serum. The period and damping rates of the oscillations were determined from a best-fit damped sine wave, using LumiCycle analysis software (Fig 1C). Plots and statistical analyses were produced using GraphPad Prism (GraphPad, CA). Heat maps were generated using Cluster 3.0 and Java Treeview software (developed by Dr. Michael Eisen while at Stanford University) (Fig 2E and 2F). For heat map plots, data were coded as positive (magenta) or negative (green) relative to the average brightness for the entire time course.

## Results

### β-adrenergic stimulation amplifies PER2 circadian rhythms in ventricle explants

To test whether manipulating myocardial function affects circadian rhythms in cardiac ventricle explant cultures, we applied isoproterenol, which is known to increase the force and rate of myocardial contractions. The ventricle explants exhibited clear PER2::LUC circadian rhythms in culture for at least 14 days, with an endogenous period of 24.5 ± 1.2 h (mean ± SD; n = 6) (Fig 1A). After 7 days of recording, the ventricle slices were treated with isoproterenol or vehicle (final 0.1% ethanol) control (Fig 1B). Isoproterenol had no effect on the period of the circadian oscillation (Fig 1C), but significantly increased the amplitude of the oscillation (Fig 1D).

### PER2 rhythms are more coherent in ventricle explants than in dispersed cardiomyocytes

In the SCN, a specialized neuronal network synchronizes single SCN neurons and reinforces rhythmicity [38]. To test whether intercellular communication plays a similar role to synchronize circadian rhythms of cardiomyocytes in ventricle explants, we imaged PER2::LUC oscillations of dispersed cardiomyocytes and compared them to rhythmicity in ventricle explants.

Cardiomyocytes were dissociated and enriched to approximately 75%-80% of the population (S1 Fig). Single beating cells were evident in bright field microscopy (S1 Movie), and cardiomyocytes could be readily distinguished from fibroblasts by the regular GCaMP3 Ca^2+^ fluorescence ‘flashes’ as they beat (S2 Movie). We imaged PER2::LUC oscillations in dispersed cardiomyocytes and ventricle explants on a microscope for 6–7 days. Thirteen of 16 beating cardiomyocytes examined in 2 cultures showed robust PER2::LUC oscillations (*p* < 0.001, chi-square periodogram) (Fig 2A and 2B). There was no statistically significant clustering of phases of dispersed cardiomyocytes within the same cultures (*p* > 0.05, Rayleigh test) (Fig 2G).

We examined four ventricle explants, two of which were contracting autonomously after serum-shock (Fig 2C, 2D, 2F and 2H, slices 1 and 2), and two of which were static without serum-shock (Fig 2F, slices 3 and 4). Flashes of GCaMP3 fluorescence accompanied contractions of explants. The ventricle explants were imaged over the course of 6–7 days. All whole explants showed robust PER2::LUC circadian rhythms (*p* < 0.001, chi-square periodogram) (Fig 2D and 2F). Damping of rhythms occurred, likely due at least in part to desynchronization among cells [32], but low amplitude oscillation was still evident even up to 7 days.

Precise delineation of single cells in ventricle explants was impractical, due to the high density of the cells (Fig 3A). Furthermore, it was difficult to identify specific ventricle tissue architecture, and so regions of interest (ROIs) of a size similar to single cardiomyocytes in dispersed cardiomyocyte cultures were generated randomly throughout the explants. As for ventricle explants, all individual ROIs showed robust PER2::LUC circadian rhythms (*p* < 0.001, chi-square periodogram) (Fig 2C and 2F). Circadian rhythms of ROIs within explants showed significant clustering of initial phases (*p* < 0.01, Rayleigh test) (Fig 2H) in both beating and non-beating explants.

The endogenous circadian period of PER2::LUC rhythms was 23.5 ± 2.2 h in dispersed cardiomyocytes (mean ± SD; n = 13 cardiomyocytes in 2 cultures) and 23.9 ± 1.8 h in ventricle explants (mean ± SD; n = 28 ROIs in 4 ventricle slices). However, *within* individual cultures, dispersed cardiomyocytes showed a much wider range of periods than did ROIs in ventricle explants (Fig 2I). The average absolute deviation from mean period *within* a culture was significantly greater in dispersed cardiomyocytes (1.7 ± 1.3 h, mean ± SD, n = 13) than in ventricle explants (0.4 ± 0.5 h, mean ± SD, n = 28 ROIs in 4 explants; *p* < 0.01, *t*-test).

### Simultaneous long-term recording of PER2 gene expression, basal Ca^2+^ level, and contraction rate in cultured ventricle explants

Previous work demonstrated that disruption of the circadian clock within cardiomyocytes influences myocardial contractile function [39]. However, functional circadian rhythms intrinsic to the heart remain largely unexplored. We wanted to determine whether there are intrinsic circadian rhythms in contraction rate or basal Ca^2+^ levels, and if so how they might be related to circadian oscillations of PER2::LUC in cultured ventricle explants. Here, we report development of a method to monitor these three parameters simultaneously and preliminary results of applying this method. Clock gene expression was monitored by PER2::LUC bioluminescence as described above. Basal Ca^2+^ level was monitor by summed GCaMP3 fluorescence level for 1 s (fluorescent image with 1 s exposure time). Contraction rate was monitored by fast (24 frames/s) GCaMP3 fluorescence stream video for 8 s. These three steps were repeated every 30 min for 6–7 d.

Simultaneous monitoring of PER2::LUC, basal Ca^2+^ levels (Fig 3A), and contraction rate (Fig 3B and 3C) was successfully performed in ventricle explants. During recording of an explant over 7 d, the rate of autonomous contractions fluctuated from 0 to 44 beats per 8 s interval (Figs 3B and 3C; and 2C, 2D, 2F and 2H, slice 1). However, there was no statistically significant circadian rhythmicity in the contraction rate (*p* > 0.05, chi-square periodogram) (Fig 3B). In two examined autonomously beating ventricle explants, the Ca^2+^ level over 7 d showed basal fluctuations as well as acute Ca^2+^ surges associated with contractions (Fig 3D). The processed Ca^2+^ data (ratio of fluorescence change to initial value, detrended by subtracting 24-h running average) did show weak circadian rhythmicity (*p* < 0.01, chi-square periodogram) in two beating ventricle explants (Fig 3E). However, these fluctuations were small (~4–5% of initial fluorescence), which is not much greater than fluctuations observed even in the absence of an explant or cells (1.7 ± 0.2%, n = 4).

To eliminate spikes in Ca^2+^ associated with contractions, imaging was performed in two non-beating ventricle explants (Figs 3F, 3G and 2H, slices 3 and 4). The time series of stream videos confirmed that there were no contractions over the 6–7 d of recording. Processed Ca^2+^ data showed weak circadian rhythmicity in one explant (Fig 3G) but not in the other (Fig 3E) (*p* < 0.01, *p* > 0.05, chi-square periodogram, respectively). However, the observed fluctuations were only 1–3% of initial fluorescence values, similar to background fluctuations in the absence of an explant or cells. In contrast, much larger fluctuations of GCaMP3 fluorescence (11.8 ± 2.9% of initial fluorescence, n = 4 slices, mean ± SEM) were seen in neonatal SCN cultured under similar conditions.

Simultaneous monitoring of PER2::LUC and basal Ca^2+^ levels was also performed in dispersed cardiomyocyte cultures. Of 8 cardiomyocytes monitored in 2 cultures over 7 d, 6 cells showed robust PER2::LUC circadian rhythms (*p* < 0.001, chi-square periodogram). As for ventricle explants, there were small fluctuations of processed basal Ca^2+^ levels that showed weak circadian rhythmicity (2 cells with *p* < 0.001, 5 cells with *p* < 0.01, 1 cell with *p* < 0.05, chi-square periodogram) (Fig 4).

## Discussion

Potential interactions between the circadian clock and Ca^2+^ in the heart are of great interest, as they may provide insight into why certain cardiovascular events are unevenly distributed throughout the 24-hour day, and why certain drug treatments are more effective when administered at a specific time of day [40, 41]. It is therefore of vital importance to determine the relationship between intrinsic myocardial circadian rhythms and myocardial functions. In this study, we showed that (1) isoproterenol increases myocardial PER2 circadian rhythm amplitude, (2) tissue organization and cellular interactions preserved in ventricle explants are sufficient to synchronize circadian rhythms of individual cardiomyocytes, and (3) cardiomyocytes exhibit robust cell-autonomous circadian rhythms of PER2 expression and weaker circadian rhythms of basal Ca^2+^, but no circadian rhythms of contraction rate. We also demonstrated a system to monitor clock gene expression, basal Ca^2+^, and contraction rate simultaneously in ventricle explants or dispersed cardiomyocytes.

Plasma cortisol and catecholamine levels show diurnal patterns, and their peaks coincide with the active phase of the animal (i.e. they peak in the morning in diurnal mammals such as humans [42], but in the evening or at night in nocturnal animals such as rodents [43]). Isoproterenol, like adrenaline, is a β1- and β2-adrenoreceptor agonist and positively modulates contraction of cardiac tissues through cAMP signaling. We show that isoproterenol, which is used to treat various heart problems including bradycardia (slow heart rate), increases the amplitude of PER2 circadian rhythms intrinsic to the heart. In a prior study, Durgan et al. [20] transiently treated isolated rat cardiomyocytes with norepinephrine, and showed an acute induction of *Bmal1* transcript followed by similar increases in *Per2*, *Dbp* and *Rev-erb α*, thereby acting a synchronizing stimulus for the cardiac circadian clock. In our study, isoproterenol induced a rapid increase in amplitude, likely through activation of cAMP signaling. Adrenal glucocorticoids are thought to be among the most important synchronizers of peripheral organ circadian rhythms [44, 45]. Glucocorticoid treatment increases both PER2 expression and rhythm amplitude in many peripheral tissues, including heart [21, 46, 47]. Further, direct activation of cAMP signaling by forskolin induces circadian clock genes in multiple cell types [48–50], and application of a cAMP analogue induces phase shifts in rat SCN explants [51]. Thus, daily peaks of adrenal catecholamines (via cAMP signaling) and cortisol likely both reinforce the intrinsic circadian oscillations of the heart. Further studies more systematically exploring effects at various circadian phases would provide a fuller understanding of how circadian rhythms of cardiomyocytes are controlled by catecholamines and glucocorticoids.

There have been few previous studies of circadian rhythms in individual cardiomyocytes, due to the difficulty of maintaining adult cardiomyocytes in long-term dispersed culture and reliably discriminating them from other cells, mainly fibroblasts [52]. We overcame these technical obstacles by culturing neonatal cardiomyocytes and using their inherent beating to identify them.

It is well established that individual neurons of the SCN, in dispersed culture, show independent oscillations in rate of spontaneous action potentials [53]. Such cell-autonomous circadian oscillations can also be detected using the PER2::LUC reporter in SCN neurons [38, 54, 55], as well as in fibroblasts [32], in which rhythms are maintained for over 40 days [56]. Early findings of cardiac circadian rhythms came from *in vivo* tissue gene expression microarray data [12, 13], which did not exclude rhythmic systemic signals originating from the SCN. A few previous studies have examined clock gene expression in heart explants or in dispersed cardiomyocytes, although not at the single-cell level [4, 20, 21]. In atrial tissue, Van der Veen et al. [21], showed phase-dependent phase-shifting of circadian rhythms in response to glucocorticoid stimulation or medium change. Response to medium change, seen in atrium but not liver, raises the possibility of specific mechanically induced effects on circadian rhythms within the heart [21]. In our study, we found consistent PER2::LUC rhythms in ventricle explants and dispersed single cardiomyocytes, which were maintained in culture over 7 days in the absence of systemic signals. These results show that single-cell cardiomyocytes are autonomous circadian oscillators, like SCN neurons and fibroblasts [53, 56].

In the SCN, specialized neuronal and paracrine communication synchronizes and strengthens circadian oscillations of individual cells [38, 57–59]. In contrast, it is thought that synchronization of cellular circadian oscillators within peripheral tissues relies primarily on synchronizing signals emanating from the SCN central pacemaker. Co-culture of fibroblasts with different circadian periods found no evidence of synchronization among fibroblasts [60–62], although a tonic level of paracrine signaling from neighbouring cells is necessary to maintain robust circadian rhythms [61]. Long-lasting circadian oscillations have been recorded in peripheral tissues cultured in isolation from the SCN [31], and weak local coupling has been observed in cultured mouse hepatocytes [63]. However, there is so far no convincing evidence of circadian synchrony mediated by direct cellular interactions within peripheral tissues. The heart, however, has a highly organized tissue structure and gap junctional coupling between cells that allow for coordinated propagation of electrical signals and contractile activity [64]. Intriguingly, we found that PER2 circadian rhythms within ventricle explants were better synchronized than those of dispersed cardiomyocytes, as evidenced by narrower initial phase and period distributions. These results suggest the presence of cellular interactions in the preserved tissue organization of the explants that contribute to circadian synchrony. We acknowledge that the number of explants was small, and results must therefore be interpreted with caution. Also, we cannot exclude the possibility that this clustering of rhythm phases and periods in explants is partly due to close packing of cells and unavoidable inclusion of multiple cells in ROIs; this issue could be addressed more definitively in future experiments using transgenic animal models or a gene gun [30] to achieve reporter expression in a scattered subset of cells, thus allowing cleaner discrimination of single cells in ROIs. Synchronized oscillations were observed in both beating and non-beating ventricle explants, which is consistent with previous work indicating that circadian period and heart rate are controlled independently [65]. After a few days, there was marked damping of rhythms in ventricle explants, probably reflecting gradual loss of synchrony among cells, which might be due to absence of the specialized conduction system mediating organized contraction in the intact heart [64]. Taken together, these results suggest that intercellular connections can at least temporarily maintain synchronized circadian oscillations within the heart, which together with daily external stimuli such as humoral and sympathetic signals, contribute to the maintenance of robust circadian rhythmicity.

Cardiomyocytes show complex and rapid Ca^2+^ dynamics that are important for their contractile function [66]. Modern fluorescent Ca^2+^ reporters have facilitated the study of rapid Ca^2+^ transients in the heart. Tallini et al. [27] used the GFP-based GCaMP2 reporter to analyse the rapid dynamics of Ca^2+^ in mouse embryonic hearts, including oscillations associated with spontaneous contractions of the isolated, perfused heart. Much slower circadian Ca^2+^ oscillations have been reported in the SCN [67, 68], as well as in plants [69]. In the SCN, Ca^2+^ is under circadian control, even independently of neuronal firing, as application of tetrodotoxin eliminates action potentials but does not affect the circadian oscillation of Ca^2+^ [30]. The expression of ion channels has also been studied extensively in the heart, with specific regions of the heart expressing different channels at varying levels of expression [70]. Some of these, including Ca^2+^ channels [23], are expressed with a circadian rhythm.

The unusually high amplitude and robustness of the SCN circadian oscillator may be related to mutual reinforcement among rhythms of neuronal firing, Ca^2+^, and clock gene transcription [71]. With each beat, the heart generates spontaneous action potentials that propagate among heart cells because they are electrically coupled through gap junctions [72–75]; it has complex, rapid Ca^2+^ dynamics; and it shows prominent circadian rhythms of clock gene expression. Also, Collins and Rodrigo found that intracellular Ca^2+^ level is about 30% higher in rat cardiomyocytes isolated during the resting period than during the active period [28]. We therefore hypothesized that the heart, like the SCN, might exhibit large, undamped oscillations in basal Ca^2+^ level and/or contraction rate. However, using the GCaMP3 reporter, we did not detect large circadian oscillations of either basal Ca^2+^ levels or contraction rate in ventricle explants. We observed no oscillations of contraction rate and only weak circadian oscillations of basal Ca^2+^ level, which were not much larger than background fluctuations (~1–5% of initial fluorescence). Similar weak oscillations in basal Ca^2+^ level were observed in single-cell dispersed cardiomyocytes. Although these weak oscillations reached statistical significance, we cannot rule out artifactual causes, such as fluctuations in LED light strength or GCaMP3 expression. In contrast, much larger fluctuations of GCaMP3 fluorescence are seen in neonatal SCN cultured under similar conditions. Further studies using larger sample size and more sensitive reporters such as aequorin [69] or newer genetically encoded Ca^2+^ indicators [76] may provide more secure evidence of circadian Ca^2+^ oscillations in the heart.

Although we did not observe strong intrinsic Ca^2+^ circadian rhythms in cultured cardiomyocytes, multiple studies suggest that dysregulation of circadian rhythms of intracellular Ca^2+^ *in vivo* could cause heart disease. Collins and Rodrigo [28] detected diurnal variations in Ca^2+^ transients in response to β-adrenergic stimulation, in ventricular myocytes freshly isolated from rat hearts. Also, they determined that there is diurnal variation in percentage of myocytes that develop arrhythmias in response to β-adrenergic stimulation, yet without changes in relevant gene transcription: L-type Ca^2+^ channel (cacna1c) or β1-adrenoreceptor [28]. Prolonged activation of one of the major Ca^2+^ regulators, calcineurin, can lead to hypertrophic remodeling of the heart, heart failure, and death [77, 78]. One of the regulators of calcineurins (RCAN) proteins that bind to and inhibit activation of calcineurins, RCAN1.4, is under circadian control [79] and is likely to be the key protein in the protection against cardiac damage from ischemia/reperfusion, which is greatest around the transition from sleep to activity [78]. These data suggest that disruption of an intricately controlled balance of oscillating Ca^2+^ and downstream signaling *in vivo* can result in cardiac failure and death.

The importance of circadian rhythm regulation intrinsic to the heart has been shown using cardiomyocyte-specific clock gene manipulations in mice. Cardiomyocyte-specific *Clock-*mutant mice lack diurnal oscillations in expression of VGCC1α1D and in phosphorylation of several kinases (e.g. ERK) [23]. In a cardiomyocyte-specific inducible *Bmal1* knock-out mouse, observed abnormalities include a slower heart rate, increased arrhythmias, and loss of circadian expression of the voltage-gated sodium channel NA_v_1.5, as well as disrupted circadian expression of the potassium channel Kcnh2, which underlies the rapidly activating delayed-rectifier K^+^ current, and contributes to normal ventricular repolarization [25]. These mice also show abnormal glucose utilization and early mortality, as a result of aged-onset cardiomyopathy [80]. A recent study of these mice has highlighted disruption to proteins involved in extracellular matrix remodelling and inflammation, together with cardiac hypertrophy and diastolic disruption [81]. These data suggest the importance of heart-specific circadian clock gene expression in modulation of ion channels, cell structure, and metabolism in the heart.

In conclusion, we have shown that adrenergic signaling amplifies circadian rhythms of the heart; that intercellular communication within ventricle explants synchronizes not only contractions but also circadian rhythms; and that simultaneous monitoring of basal Ca^2+^ level, contraction rate, and clock gene expression is possible in cardiomyocytes. Further studies using genetically encoded Ca^2+^ indicators with a broader dynamic range may provide further insights into the intrinsic circadian properties of myocardial functions. There is a wealth of accumulating evidence linking the circadian clock to many aspects of heart disease [82–85]. The current data only begin to define the relationship between cardiac structure, function, and circadian biology [12, 13, 21, 86, 87]. A thorough understanding of the relationship between the circadian clock and Ca^2+^ in the heart, at the single-cell and tissue levels, may lead to medical interventions that complement, rather than oppose, rhythmic biological processes.

## Supporting Information

S1 Fig Immunofluorescence imaging of alpha-actinin in primary cardiomyocytes. Primary cardiomyocytes were isolated from 2 day old mouse pups and labelled with an antibody to alpha-actinin (A) and DAPI (B). A merged image is shown below (C). Scale bar is 250 μm.
(TIF)

S1 Movie Beating of a single cardiomyocyte in dispersed culture. Myocardial contractions in single cardiomyocytes revealed by bright field imaging.
(MP4)

S2 Movie Ca^2+^ surges associated with contraction in dispersed cardiomyocytes. Myocardial contractions in single cardiomyocytes reflected in GCaMP3 fluorescence imaging.
(MP4)
