# Supplementary figures and images for: Cardiomyocyte Circadian Oscillations Are Cell-Autonomous, Amplified by β-Adrenergic Signaling, and Synchronized in Cardiac Ventricle Tissue
Source: PLoS One. 2016 Jul 26;11(7):e0159618. doi: 10.1371/journal.pone.0159618 (PMC4961434; doi:10.1371/journal.pone.0159618)

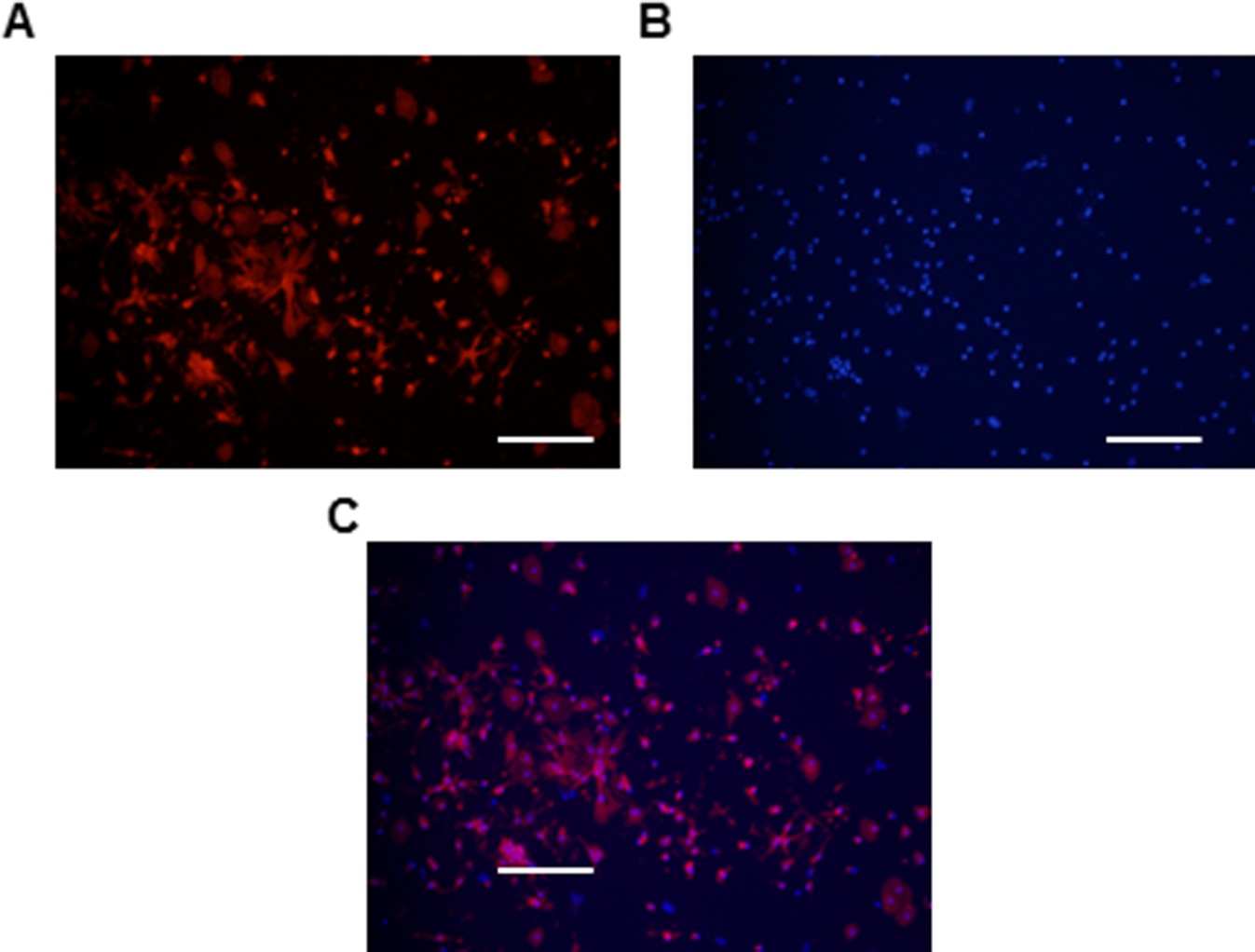

Supplement: S1 Fig — Primary cardiomyocytes were isolated from 2 day old mouse pups and labelled with an antibody to alpha-actinin (A) and DAPI (B). A merged image is shown below (C). Scale bar is 250 μm. (TIF) [file pone.0159618.s001.tif]
